# Murine and Human-Purified very Small Embryonic-like Stem Cells (VSELs) Express Purinergic Receptors and Migrate to Extracellular ATP Gradient

**DOI:** 10.1007/s12015-024-10716-4

**Published:** 2024-04-18

**Authors:** Kamila Bujko, Katarzyna Brzezniakiewicz-Janus, Justyna Jarczak, Magdalena Kucia, Mariusz Z. Ratajczak

**Affiliations:** 1 Department of Regenerative Medicine, Center for Preclinical Studies and Technology, Warsaw Medical University, Warsaw, Poland; 2 Department of Hematology, University of Zielona Gora, Multi-Specialist Hospital Gorzow Wlkp., Zielona Gora, Poland; 3 Stem Cell Institute at Graham Brown Cancer Center, University of Louisville, 500 S. Floyd Street, Rm. 107, Louisville, KY 40202 USA

**Keywords:** VSELs, Purinergic receptors, Chemotaxis, Extracellular ATP, Extracellular Ado, Nlrp3 inflammasome, MCC950

## Abstract

Purinergic signaling is an ancient primordial signaling system regulating tissue development and specification of various types of stem cells. Thus, functional purinergic receptors are present in several types of cells in the body, including multiple populations of stem cells. However, one stem cell type that has not been evaluated for expression of purinergic receptors is very small embryonic stem cells (VSELs) isolated from postnatal tissues. Herein, we report that human umbilical cord blood (UCB) and murine bone marrow (BM) purified VSELs express mRNA for P1 and P2 purinergic receptors and CD39 and CD73 ectonucleotidases converting extracellular ATP (eATP) into its signaling metabolite extracellular adenosine (eAdo), that antagonizes eATP effects. More importantly, we demonstrate that human and murine VSELs respond by chemotaxis to eATP, and eAdo inhibits this migration. These responses to eATP are mediated by activation of Nlrp3 inflammasome, and exposure of VSELs to its specific inhibitor MCC950 abolished the chemotactic response to ATP. We conclude that purinergic signaling plays an essential, underappreciated role in the biology of these cells and their potential role in response to tissue/organ injuries.

**Graphical Abstract:**

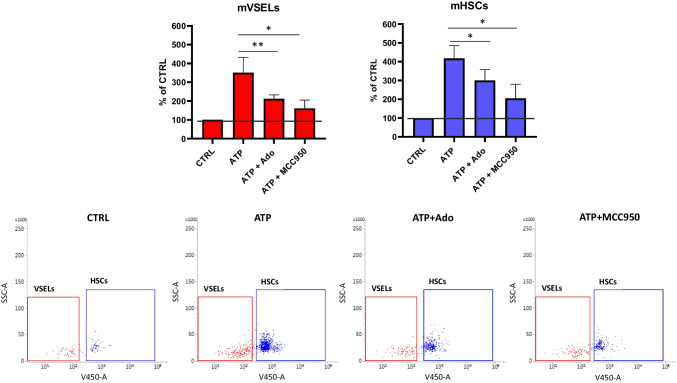

## Introduction

Purinergic signaling is involved in embryogenesis and governs the development and functioning of several tissues [1, 2]. This primordial form of extracellular signaling is mediated by extracellular nucleotides, including mainly extracellular ATP (eATP) and its nucleoside metabolite, extracellular adenosine (eAdo) [1]. While eATP engages several ionotropic P2X and G-protein coupled P2Y receptors, eAdo stimulates the family of G-protein coupled P1 receptors and inhibits several eATP-mediated responses. In addition to eATP and eAdo, other rare signaling extracellular nucleotides include some pyrimidines like UTP, UDP, or UDP-glucose. The P2X ionotropic channel receptor family stimulated exclusively by eATP consists of seven members (P2X1, 2, 3, 4, 5, 6, and 7), whereas the P2Y family responding to ATP, adenosine diphosphate (ADP), uridine triphosphate (UTP), uridine diphosphate (UDP), or UDP-glucose includes a total of eight receptors (P2Y1, 2, 4, 6, 11, 12, 13, and 14) [1–6]. The gaps in P2Y receptor numbering are because several receptors (P2Y3, P2Y5, P2Y7, P2Y8, P2Y9, and P2Y10) which have been cloned and were assigned as members of the P2Y receptor family have been removed from the list as purinergic ligands do not activate them. The P1 family activated by eAdo comprises four subtypes (A1, A2A, A2B, and A3) [1–6].

Purinergic signaling, a primordial communication system mediated by extracellular nucleotides [1, 2], affects the proliferation and specification of many adult and embryonic stem cell types. This signaling, as reported, is involved in embryogenesis and governs the development and functioning of several tissues.

Very small embryonic-like stem cells (VSELs) are a population of early-development stem cells that, as postulated, are deposited during development in several postnatal murine and human tissues and organs, including bone marrow (BM) [6–8]. These cells are also detectable in peripheral blood (PB), and their number increases in circulation in response to stress, strenuous exercise, infection, and tissue/organ injury []. This explains an increase in the number of VSELs circulating in human umbilical cord blood (UCB) collected after delivery. As reported, these small cells have broader differentiation potential across germ layers and may give rise to various populations of tissue-specific stem cells. Several independent laboratories have confirmed the presence of VSELs in postnatal murine and human tissues [8]. These discoveries challenged the view on the adult stem cell compartment hierarchy. Accordingly, as reported, VSELs may become specified, e.g., to hematopoietic cells [9], endothelial progenitors [10], and gametes [11], as well as contribute to the cardiomyocytes [12], hepatocytes [13], and pulmonary alveolar epithelium [14].

Since VSELs are early development stem cells deposited in postnatal tissues, we become interested in the expression of purinergic receptors on these cells and their responsiveness to the primary purinergic signaling ligands, eATP and eAdo.

## Material and Methods

### Isolation of Human VSELs and HSCs

**Fig. 1:**
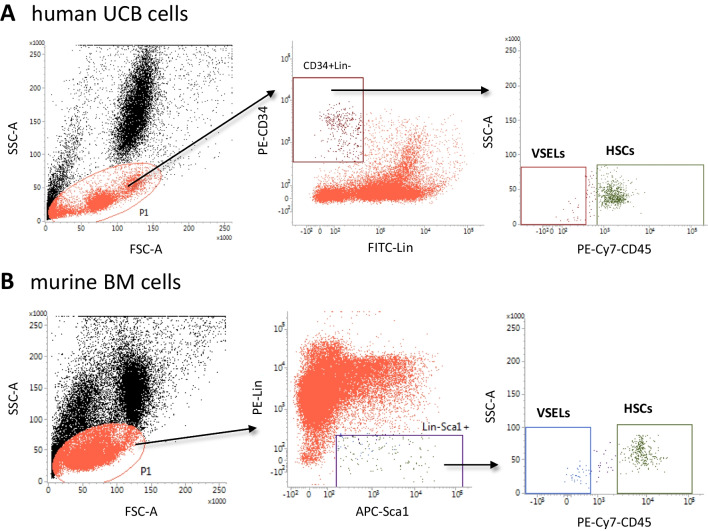
Gating strategy for sorting VSELs and HSCs by FACS. Human umbilical cord blood (hUCB) mononuclear cells (MNCs) were isolated by Ficoll gradient centrifugation. MNCs were then immunostained and sorted. Briefly, cells were visualized by dot plot showing forward scatter (FSC) vs. side scatter (SSC) signals, where small events ranging from 4–12 µm were gated (P1). Cells from region P1 were further analyzed for CD34 and Lin expression. The population of CD34^+^Lin^−^ events were then analyzed for CD45 expression and subsequently sorted as Lin^−^CD34^+^CD45^−^ VSEL and Lin^−^CD34^+^CD45^+^ HSCs subpopulations (**Panel A**). Murine bone marrow cells were isolated, and red blood cells were removed by ammonium chloride lysis. Cells were then stained and sorted. First, small cells ranging from 2 to 10 µm were included in the gate P1 and subsequently analyzed for the Sca1 and Lin markers expression. Sca1^+^Lin^−^ objects were further analyzed for CD45 antigen expression and sorted as Sca1^+^Lin^−^CD45^−^ VSEL and Sca1^+^Lin^−^CD45^+^ HSC subpopulations (**Panel B**). Representative dot plots are shown

Human umbilical cord blood (hUCB) was obtained from healthy newborns delivered at the Department of Obstetrics and Gynecology, Medical University of Warsaw (Warsaw Bioethics Committee permission number KB/3/2018). hUCB units, containing a minimum of 20 ml, were diluted with phosphate-buffered saline (PBS) and carefully layered over Ficoll-Paque (GE Healthcare, Chicago, IL, USA) and centrifuged for 30 min at 400 × g at 4 °C. The lymphocytes/monocytes/platelets phase was collected, washed, and used for further analysis. Briefly, cells were stained with the following antibodies: hematopoietic lineage markers (Lin) cocktail of antibodies, each FITC-conjugated: CD235a (clone GA-R2 [HIR2]), anti-CD2 (clone RPA-2.10), anti-CD3 (clone UCHT1), anti-CD14 (clone M5E2), anti-CD16 (clone 3G8), anti-CD19 (clone HIB19), anti-CD24 (clone ML5), anti-CD56 (clone NCAM16.2) and anti-CD66b (clone G10F5) (all BD Biosciences, San Jose, CA, USA); PE-Cy7-conjugated anti-CD45 (clone HI30, BioLegend, San Diego, CA, USA) and PE-conjugated anti-CD34 (clone 581, BioLegend, San Diego, CA, USA). Antibodies were used in the manufacturer’s recommended concentration. Cells were stained in the dark at 4 °C for 30 min, then centrifugated and resuspended in RPMI-1640 medium containing 2% fetal bovine serum (FBS, Corning Inc, Corning, NY, USA). Cells were sorted according to the strategy shown in Fig. 1A. Briefly, small events (4–12 μm in size) were included in the “lymphocyte-like” gate and then further analyzed for the expression of Lin marker, CD45, and CD34 antigens (Fig. 1). Populations of VSELs (Lin^−^CD45^−^CD34^+^) and HSCs (Lin^−^CD45^+^CD34^+^) were sorted on the MoFlo Astrios EQ cell sorter (Beckman Coulter, Brea, CA, USA).

### Isolation of Murine VSELs and HSCs

Animal studies were approved by the Animal Care and Use Committee of the Warsaw Medical University (Warsaw, Poland). Pathogen-free, 6–8-week-old C57BL/6 J wild-type (WT) mice were purchased from the Central Laboratory for Experimental Animals, Medical University of Warsaw. Bone marrow (BM) VSELs and HSCs were isolated from sacrificed WT mice. Briefly, cells were flushed from the tibias and femurs with cold PBS. Next, Red Blood Cells (RBCs) were lysed using 1 × BD Pharm Lyse buffer (BD Pharmingen, San Jose, CA, USA). Obtained population of cells (MNCs + granulocytes) was washed and resuspended in RPMI-1640 medium (Corning Inc, Corning, NY, USA) containing 2% FBS (Corning Inc, Corning, NY, USA) and subsequently stained with the following anti-mouse antibodies: Lin antibodies cocktail, all PE, rat anti: CD45R/B220 (clone RA3-6B2), Gr-1 (clone RB6-8C5), TCRαβ (clone H57-597), TCRγδ (clone GL3), CD11b (clone M1/70), Ter119 (clone TER-119) (BD Pharmingen, San Jose, CA, USA); PE-Cy7 anti-CD45 (clone 30-F11) (BD Pharmingen, San Jose, CA, USA) and APC Ly-6A/E (Sca1) (clone E13-161.7) (BioLegend, San Diego, CA, USA). The cells were then washed and resuspended in RPMI-1640 medium plus 2% FBS, and the population of VSELs (Lin^−^Sca1^+^CD45^−^) and HSCs (Lin^−^Sca1^+^CD45^+^) were sorted using the MoFlo Astrios EQ cell sorter (Beckman Coulter, Brea, CA, USA) accordingly to strategy shown on the Fig. 1B.

### Quantitative RT-PCR

Total RNA from murine and human VSELs, HSCs, and MNCs was isolated using RNeasy Micro and RNeasy Mini kit (Qiagen Inc., Hilden, Germany). RNA concentration was measured on NanoDrop, and at least 100 ng of RNA was reverse transcribed with the iScript reverse transcription kit (Bio-Rad, Hercules, CA, USA). The target genes were evaluated using iTaq Universal SYBR Green Supermix (Bio-Rad, Hercules, CA, USA) and specific primers listed in Tables 1 and 2. The samples were run on the CFX384 Touch™ Real-Time PCR detection system (Bio-Rad, Hercules, CA, USA). The PCR cycling conditions were 95 °C (30 s), 45 cycles at 95 °C (15 s), and 60 °C (30 s). A melting curve was created to emphasize the specificity of the primer and avoid the possibility of amplifying DNA contamination. Quantification was calculated using the comparative ΔCT method where mRNA levels of target receptors were normalized to the β-2microglobulin (human samples) or β-actin (murine samples) mRNA level. MNCs were assigned as a control group. According to melting point analysis, only one PCR product was amplified under these conditions. PCR products were visualized on 2% agarose gels.

**Table 1:** Sequenes of the human primers

Human gene	Forward primer	Reverse primer
*β-2MG*	TGGGTTTCATCCATCCGACA	TCAGTGGGGGTGAATTCA
*CD39*	TCGCCTCTATGGCAAGGACTAC	TCCAGGATGAAAGCATGGGTCC
*CD73*	AGTCCACTGGAGAGTTCCTGCA	TGAGAGGGTCATAACTGGGCAC
*A1*	TGCGAGTTCGAGAAGGTCATC	GAGCTGCTTGCGGATTAGGTA
*A2a*	CGAGGGCTAAGGGCATCATTG	CTCCTTTGGCTGACCGCAGTT
*A2b*	TAAAAGTTTGGTCACGGGGACCCGA	TTCACAAGGCAGCAGCTTTCATTCGT
*A3*	TACATCATTCGGAACAAACTC	GTCTTGAACTCCCGTCCATAA
*P2X1*	CCTCATCAGCAGTGTCTCTG	GGTCATGACCACGAAGGAGT
*P2X2*	GTGGAAATGAAAGACATCATCGTGCTGGT	AGGCCCAGGAGGAATCTGAATGGG
*P2X3*	GGTTTTCTTGCACGAGAAGGCTTACCA	TGAGGTGGCGTCACGTAATCAGACA
*P2X4*	CCTCTGCTTGCCCAGGTACTC	CCAGGAGATACGTTGTGCTCAA
*P2X5*	CTGCCTGTCGCTGTTCGA	GCAGGCCCACCTTCTTGTT
*P2X6*	AGGCCAGTGTGTGGTGTTCA	TCTCCACGGGGCACCAACTC
*P2X7*	AGTGCGAGTCCATTGTGGAG	CGCAGGTCTTGGGACTTCTT
*P2Y1*	GCCATCTGGATGTTCGTCTTCC	TGGCAGAGTCAGCACGTACAAG
*P2Y2*	CGAGGACTTCAAGTACGTGCTG	GTGGACGCATTCCAGGTCTTGA
*P2Y4*	TGCCTGGTCACTCTTGTTTG	GTACTCGGCAGTCAGCTTCC
*P2Y6*	AACCGCACTGTCTGCTATGACC	AGCAGGAAGCCGATGACAGTGA
*P2Y11*	AGGGCAAAGTGATGTTCCAC	CCCTCCAGGCTCTTCTTTCT
*P2Y12*	AACTGGGAACAGGACCACTG	ACATGAATGCCCAGATGACA
*P2Y13*	GCCGACTTGATAATGACACTCATG	CCTAACAGCACGATGCCCACAT
*P2Y14*	GCCGCAACATATTCAGCATCGTG	GCTGTAATGAGCTTCGGTCTGAC

**Table 2:** Sequenes of the murine primers

Murine gene	Forward primer	Reverse primer
*β-actin*	ACCCCAGCCATGTACGTAGCCAT	CAGGATGGCGTGAGGGAGAGCAT
*CD39*	CTGGACAAGAGGAAGGTGCCTA	GACTGTCTGAGATGAGGCTTAGC
*CD73*	CGCTCAGAAAGTTCGAGGTGTG	CGCAGGCACTTCTTTGGAAGGT
*A1*	TTGTGGTAGGCCTGACACCCATGT	GCCGTTGGCTATCCAGGCTTGTT
*A2a*	CGAAGGGCATCATTGCGATTTGCTG	ATGTAGGAAAAGACGATCATGTGCAAGACC
*A2b*	TGCATTACAGACCCCCACCAACTACTTT	AAGAGGCTAAAGATGGAGCTCTGTGTGAG
*A3*	CTGGCCATTGCTGTAGACCG	GTCAGCCCCACCAGAAAGGA
*P2X1*	CCAGACCTCAAGTGGCCTTATCAGC	CTGGGAAGACATAGTCAGCCACGTC
*P2X2*	GGCGGTGTCATTGGGGTCATCAT	AAGAGGCAGGGTCATACTTGGGGT
*P2X3*	TCTCCAGCAGAGACATCAGCA	GGAGCATCTTGGTGAACTCAG
*P2X4*	CATTTATAATGCGCGGACGGATCCCT	CTCCACTGCCATCTCCTGAAAGCTG
*P2X5*	GGAAGATAATGTTGAGGTTGAG	TCCTGATGAACCCTCTCCAGT
*P2X6*	CCCAGAGCATCCTTCTGTTCC	GGCACCAGCTCCAGATCTCA
*P2X7*	CAGTATGAGACAAACAAAGTCACCCGGA	ATGTAGGAAAAGACGATCATGTGCAAGACC
*P2Y1*	CCTGCTATGACACCACGTCCAA	AGCGGAGAGTTGTCCAGGTCAT
*P2Y2*	TTCACCTGGCAGTTTCGGACTC	GTGTAGAAGAGGAAACGCACCAG
*P2Y4*	CTGGACAGTCATCTTCTCGGCT	TTCGGCGTTCAACAGTCTTGCC
*P2Y6*	CAGTCTTTGCTGCCACAGGCAT	AGCAAGAAGCCGATGACCGTGA
*P2Y10*	GAGCCAGAAACTGGAAGCGTAG	GGCTAAGCCAGCATTTCTCAGG
*P2Y12*	CATTGACCGCTACCTGAAGACC	GCCTCCTGTTGGTGAGAATCATG
*P2Y13*	TGGCATCAGGTGGTCAGTCACA	TTGTGCCTGCTGTCCTTACTCC
*P2Y14*	ACCTCCGTCAAGAGGAAGTCCA	GCTGTAGTGACCTTCCGTCTGA

### Isolation of Sca1^+^ Cells from Murine Bone Marrow

Pathogen-free, 6–8-week-old C57BL/6 J WT mice were purchased from the Central Laboratory for Experimental Animals, Medical University of Warsaw. Mice were sacrificed, and cells were flushed from the tibias and femurs with cold PBS. Next, RBCs were lysed using 1 × BD Pharm Lyse buffer (BD Pharmingen, San Jose, CA, USA). Subsequently, BM cells were labeled with the Anti-Sca-1 MicroBead Kit (Vio® Bright FITC) (Miltenyi Biotec, Bergisch Gladbach, Germany) according to the manufacturer’s protocol. Sca1^+^ cells were then isolated on a QuadroMacs separator with the use of LS columns (both Miltenyi Biotec and Bergisch Gladbach, Germany).

### Isolation of CD34^+^ Cells from Human Umbilical Cord Blood

hUCB was obtained from healthy newborns delivered at the Department of Obstetrics and Gynecology, Medical University of Warsaw. hUCB units, containing a minimum of 20 ml, were diluted with PBS and carefully layered over Ficall-Paque (GE Healthcare, Chicago, IL, USA) and centrifuged for 30 min at 400 × g at 4 °C. Phase containing MNCs was collected, and cells were then washed and labeled with anti-CD34 beads using a CD34 MicroBead Kit (Miltenyi Biotec, Bergisch Gladbach, Germany). CD34^+^ cells were isolated on a QuadroMacs separator with the use of LS columns (both Miltenyi Biotec and Bergisch Gladbach, Germany).

### Transwell Migration Assay

Cell migration was evaluated using the Boyden Chamber system. Murine Sca1^+^ or human CD34^+^ cells were starved in RPMI-1640 medium containing 0.5% bovine serum albumin (BSA, Sigma-Aldrich, Saint Louis, MO, USA) for 1 h or with 0.5% BSA RPMI-1640 containing 10 μM MCC950 (MedChemExpress, Princeton, NJ, USA). Lower chambers of a Costar Transwell 24-well plate (Corning Inc, Corning, NY, USA) (min. two wells per group) were pre-filled with 0.5% BSA RPMI-1640 for control or with 10 μM adenosine triphosphate (ATP, Sigma-Aldrich, Saint Louis, MO, USA) or 10 μM ATP with 10 μM Adenosine (Ado, Sigma-Aldrich, Saint Louis, MO, USA) or with 10 μM ATP and 10 μM MCC950. Cells were loaded onto the inserts (6.5 mm diameter membrane with 5 µm pores, Corning Inc, Corning, NY, USA) where at least 3 × 10^5^ Sca1^+^ or 5 × 10^4^ CD34^+^ cells were applied. The inserts were placed on a 24-well plate. The plate was incubated for 3 h at 37 °C in a 5% CO_2_ incubator. Following incubation, medium from the lower chamber containing migrated cells was harvested and centrifuged. Cells were resuspended in 2% FBS RPMI-1640 and stained with the following antibodies: murine cells – Lin cocktail (described above), APC anti-Sca1 (clone E13-161.7) and V450 anti-CD45 (clone 30-F11) (BD, Biosciences, San Jose, CA, USA); human – Lin cocktail (described above), PE anti-CD45 (clone HI30, BD Biosciences, San Jose, CA, USA) and APC anti-CD34 (clone 581, BD Biosciences, San Jose, CA, USA). Samples were analyzed on FACS Verse (BD Biosciences, San Jose, CA, USA) where murine VSELs (Lin^−^Sca1^+^CD45^−^), HSCs (Lin^−^Sca1^+^CD45^+^), and human VSELs (Lin^−^CD34^+^CD45^−^) and HSCs (Lin^−^CD34^+^CD45^+^) were counted.

### Statistical Analysis

Statistical analysis was performed using GraphPad Prism 9.0 (GraphPad Software Inc). All data are provided as an average ± SD. Statistical data analysis was performed using multiple unpaired t-tests with Welsh correction. In all calculations, *p* < 0.05 was considered significant.

## Results

### Purification of Human UCB- and Murine BM-Derived VSELs and HSCs by FACS

Human umbilical cord blood (hUCB) mononuclear cells (MNCs) were isolated by Ficoll gradient centrifugation, stained, and sorted by FACS as described above. Figure 1A demonstrates a dot plot showing forward scatter (FSC) vs. side scatter (SSC) signals, where small events ranging from 4–12 µm were gated (P1). Cells from region P1 were further analyzed for CD34 antigen and lineage (Lin) marker expression. The population of CD34^+^Lin^−^ events were then analyzed for the presence of CD45 and subsequently sorted as Lin^−^CD34^+^CD45^−^ VSEL and Lin^−^CD34^+^CD45^+^ HSCs subpopulations [8]. Figure 1B shows the purification of murine BM-derived VSELs. Murine BM cells were isolated, and red blood cells were removed by ammonium chloride lysis. Cells were then stained and sorted. First, small cells ranging from 2 to 10 µm were included in the gate P1 and subsequently analyzed for the Sca1 and Lin markers expression. Sca1^+^Lin^−^ objects were further analyzed for CD45 antigen expression and sorted as Sca1^+^Lin^−^CD45^−^ VSEL and Sca1^+^Lin^−^CD45^+^ HSC subpopulations [8, 9].

### Expression of Purinergic Receptors on Human Cord Umbilical Blood (UCB) Derived VSELs

**Fig. 2:**
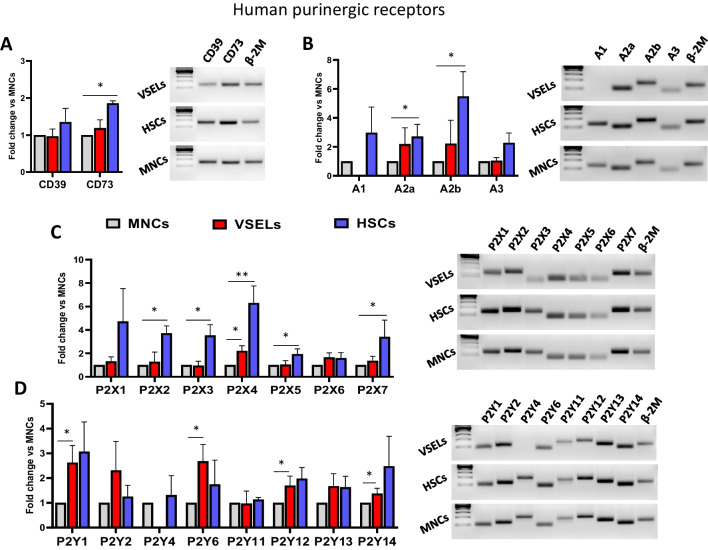
Purinergic receptors are expressed on human cord umbilical blood (hUCB) derived VSELs. mRNA was isolated from VSELs, HSCs, and MNCs sorted from hUCB as described in Materials and Methods and shown in Fig. 1A. After reverse transcription, samples were analyzed for the expression of CD39 and CD73 (**Panel A**), P1 (**Panel B**), P2X (**Panel C**) and P2Y (**Panel D**) receptors. The mRNA levels of target genes were normalized to the β-2-micoglobuline’s (β-2 M) mRNA level. The relative expression of targeted genes in VSELs and HSCs versus MNCs was calculated using the comparative ΔCT method. Results from 3–5 independent purifications from different UCBs are combined and shown as means ± SD. *p < 0.05; **p < 0.01. Additionally, products of the qRT-PCR reaction were visualized on 2% agarose gel. Representative gel pictures are shown

mRNA was isolated from VSELs, HSCs, and MNCs sorted from hUCB as described in Materials and Methods and shown in Fig. 1A. After reverse transcription, samples were analyzed for the expression of CD39 and CD73 (Fig. 2A), P1 (Fig. 2 B), P2X (Fig. 2C) and P2Y (Fig. 2D) receptors. The mRNA levels of target genes were normalized to the β-2-microglobulin (β-2 M) mRNA level. The relative expression of targeted genes in VSELs and HSCs versus MNCs was calculated using the comparative ΔCT method. Additionally, products of the qRT-PCR reaction were visualized on 2% agarose gel. Representative gel pictures are shown. Human UCB-derived VSELs expressed all the messages for purinergic receptors except mRNA for the A_1_ receptor.

### Expression of Purinergic Receptors on Murine Bone Marrow (BM) Derived VSELs

**Fig. 3:**
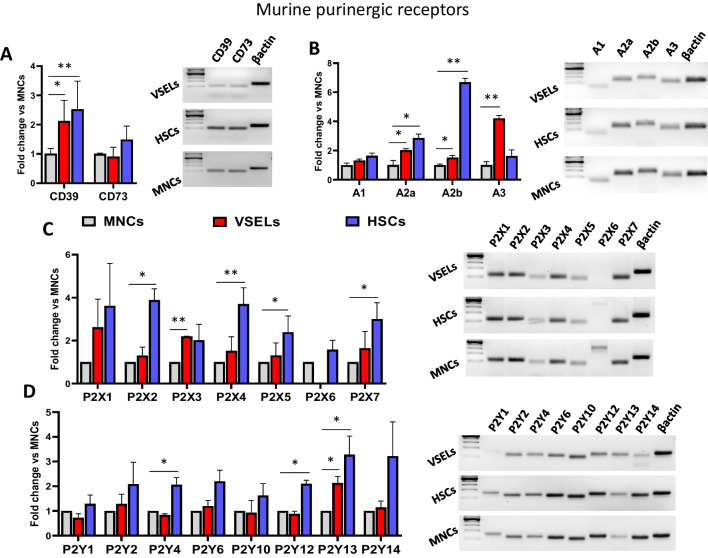
Purinergic receptors are expressed on murine bone marrow (BM) VSELs. mRNA was isolated from VSELs, HSCs, and MNCs sorted from murine BM as described in Materials and Methods and shown in Fig. 1B. After reverse transcription, samples were analyzed for the expression of CD39 and CD73 (**Panel A**), P1 (**Panel B**), P2X (**Panel C**) and P2Y (**Panel D**) receptors. The mRNA levels of target genes were normalized to the β-actin’s mRNA level. The relative expression of targeted genes in VSELs and HSCs versus MNCs was calculated using the comparative ΔCT method. Results are combined from 3 independent purifications from three animals and shown as means ± SD. *p < 0.05; **p < 0.01. Additionally, products of the qRT-PCR reaction were visualized on 2% agarose gel. Representative gel pictures are shown

mRNA was isolated from VSELs, HSCs, and MNCs sorted from murine BM as described in Materials and Methods and shown in Fig. 1B. After reverse transcription, samples were analyzed for the expression of CD39 and CD73 (Fig. 3A), P1 (Fig. 3B), P2X (Fig. 3C) and P2Y (Fig. 3D) receptors. The mRNA levels of target genes were normalized to the β-actin’s mRNA level. The relative expression of targeted genes in VSELs and HSCs versus MNCs was calculated using the comparative ΔCT method. Additionally, products of the qRT-PCR reaction were visualized on 2% agarose gel. As is shown, murine BM-derived VSELs expressed all the messages for purinergic receptors except mRNA for the P2X6 receptor. Moreover, we did not evaluate the mRNA level for the P2Y11 receptor because murine cells, unlike humans, do not express this receptor [15].

### eATP Promotes the Migration of Human Umbilical Cord Blood (hUCB)- and Murine BM-derived VSELs and HSCs

**Fig. 4:**
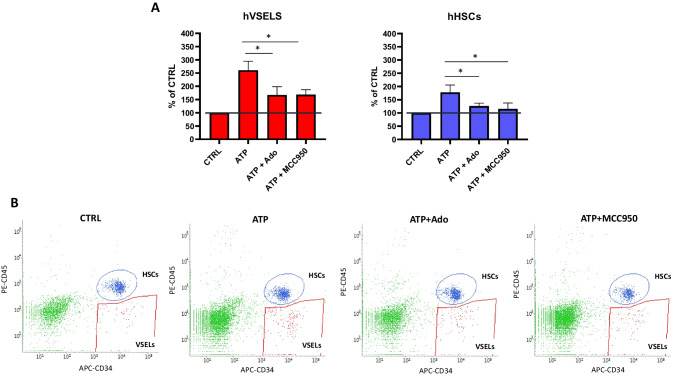
ATP promotes the migration of human umbilical cord blood (hUCB) VSELs and HSCs. The chemotactic responsiveness of hUCB VSELs and HSCs to 10 µM ATP, 10 µM ATP + 10 µM Adenosine (Ado), and 10 µM ATP + 10 µM MCC950 gradients was analyzed by FACS. CD34^+^ cells were isolated as described in Materials and Methods. Migration potential was then elucidated using the Boyden Chamber system. Cells that migrated in the gradient of used chemoattractants were harvested and immunostained. VSELs and HSCs were then enumerated using a similar gating strategy, as shown in Fig. 1A. A number of migrated VSELs and HSCs is shown as a % of the control group where no factor was used. Results are combined from three independent experiments using three different UCB samples shown as means ± SD. *p < 0.05 (**Panel A**). Representative dot plots for VSELs’ and HSCs’ enumeration (Panel B) are shown

**Fig. 5:**
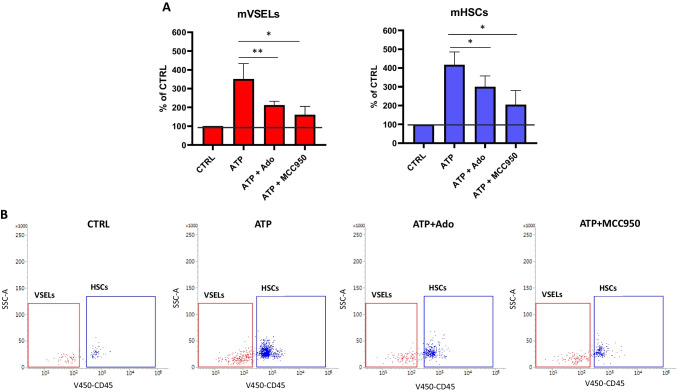
ATP promotes the migration of murine bone marrow (BM) VSELs and HSCs. The chemotactic responsiveness of murine BM VSELs and HSCS to 10 µM ATP, 10 µM ATP + 10 µM Adenosine (Ado), and 10 µM ATP + 10 µM MCC950 gradients was analyzed by FACS. Sca1^+^ cells were isolated as described in Materials and Methods. Migration potential was then elucidated using the Boyden Chamber system. Cells that migrated in the gradient of used chemoattractants were harvested and immunostained. VSELs and HSCs were then enumerated. First, small cells ranging from 2 to 10 µm were gated and analyzed for the Sca1 and Lin markers expression as shown in Fig. 1B. Then Sca1^+^Lin^−^ objects were further analyzed for CD45 marker expression and counted as Lin^−^CD34^+^CD45^−^ VSEL and Lin^−^CD34^+^CD45^+^ HSCs. A number of migrated VSELs and HSCs is shown as a % of the control group where no factor was used. Results are combined from five independent experiments using five different UCB samples as means ± SD *p < 0.05; **p < 0.01 (**Panel A**). Representative dot plots for VSELs’ and HSCs’ enumeration (**Panel B**) are shown

To address the responsiveness of human UCB-derived VSELs to eATP and eAdo, we employed the Transwell migration assay. Purified cells were loaded into the upper chamber and tested against eATP and eATP + eAdo gradients. Based on data indicating that the chemotaxis of cells to the eATP gradient is mediated by Nlrp3 inflammasome, we also employed its specific inhibitor, MCC950 (Fig. 4A). Cells that migrated in the gradient of used chemoattractants were harvested and immunostained (Fig. 4B). These experiments show that human VSELs respond to eATP gradient by migration in an Nlrp3 inflammasome-dependent manner. Similar results were obtained with murine BM-purified VSELs (Fig. 5A and B).

## Discussion

BM contains hematopoietic stem/progenitor cells (HSPCs) and several populations of non-hematopoietic stem cells, including mesenchymal (MSCs) and endothelial progenitor cells (EPCs) [16]. Nevertheless, the BM stem cell compartment still needs to be characterized better. To support this, our team described rare stem cells with broader, cross-germ layer specification potential that were named very small embryonic-like stem cells (VSELs) [6–14]. Subsequently, several independent laboratories have confirmed the presence of these cells in postnatal tissues [6–14].

The proliferation and specification of many adult and embryonic stem cell types are affected by purinergic signaling, a primordial communication system mediated by extracellular nucleotides [1, 2]. This signaling, as reported, is involved in embryogenesis and governs the development and functioning of several tissues. Because VSELs are early development stem cells deposited in postnatal tissues, we become interested in the expression of purinergic receptors on these cells and their potential responsiveness to major purinergic signaling ligands, including eATP and eAdo.

Herein, we report for the first time that VSELs isolated from human UCB and murine BM express at mRNA several purinergic receptors from the P2X ionotropic channel receptor family stimulated exclusively by eATP, P2Y receptors responding in addition to eATP to several purines (extracellular ADP) and, pyrimidines (extracellular uridine triphosphate; eUTP and, extracellular uridine diphosphate; e-UDP), and the P1 family of purinergic receptors activated by eAdo. All these receptors were expressed by purified VSELs and HSCs; however, expression tends to be higher by HSCs. Interestingly, at the same time, we noticed a lack of A1 receptor expression on human UCB-derived and P2X6 receptors on murine BM-derived VSELs.

It is well known that eATP is a potent chemoattractant for cells, including adult stem cells [17]. This has been demonstrated for HSCs [17, 18], MSCs [17], and EPCs [17]. Since VSELs express several P2X receptors, we tested their chemotactic response to eATP in the Transwell migration system and noticed that both human and murine VSELs migrated to the eATP gradient. Our data with HSPCs indicate that mainly P2X1, P2X3, P2X4, and P2X7 receptors regulate the chemotaxis of these cells to the eATP gradient [4, 5]. Since all these receptors are also expressed on human UCB- and murine BM-derived VSELs, we plan to employ receptor-specific inhibitors and receptor-deficient mice to address their role in migrating VSELs to eATP gradient.

Moreover, since the chemotactic responsiveness of several cell types to eATP is regulated negatively by eAdo and VSELs express P1 receptors, we evaluated chemotaxis to eATP alone and in the presence of added eAdo. As expected, chemotactic response was inhibited in the presence of eAdo [19]. Our previous research also demonstrated that cell migration to the eATP gradient depends on activating intracellular pattern recognition receptor—Nlrp3 inflammasome [20]. Again, human and murine VSELs exposure to Nlrp3 inflammasome inhibitor MCC950 decreased eATP-mediated migration. This confirms the critical role of Nlrp3 inflammasome in cell migration [21]. Since eATP is released from stressed and damaged cells and, similarly, activation of Nlrp3 inflammasome occurs in response to stress, infection, and tissue organ injury, our data indicate an essential role of eATP-purinergic receptors-Nlrp3 inflammasome axis signaling in attracting VSELs to mend damaged organs.

However, murine and human VSELs express mRNA for several purinergic receptors; this expression in steady-state conditions was lower in VSELs than in HSPCs. This could depend on the quiescent state of these cells. Nevertheless, in the functional studies discussed above, VSELs respond positively to a chemotactic gradient of eATP that the P2X receptor family mainly mediates, and this effect was inhibited by eAdo after activation of P1 receptors. This indicates that despite the difference in the mRNA level between HSPCs and VSELs, these receptors are functional in human and murine VSELs.

In conclusion, our data opens a new view on the role of purinergic signaling in the biology of most primitive stem cells in adult tissues and explains their potential role in tissue/organ regeneration.

## Data Availability

Detailed data is available upon request.
